# Motivational interviewing in respiratory therapy: What do clinicians need to make it part of routine care? A qualitative study

**DOI:** 10.1371/journal.pone.0187335

**Published:** 2017-10-31

**Authors:** Robert Shannon, Maggie Donovan-Hall, Anne Bruton

**Affiliations:** 1 Faculty of Health Sciences, University of Southampton, Southampton, United Kingdom; 2 Southampton NIHR Biomedical Research Centre, Southampton, United Kingdom; 3 Wessex NIHR CLAHRC, Southampton, United Kingdom; Universite de Bretagne Occidentale, FRANCE

## Abstract

Motivational interviewing (MI) is a method for building motivation for behaviour change that has potential for use in respiratory contexts. There is a paucity of published research exploring the feasibility of this intervention from the clinicians’ perspective. This study aimed to explore respiratory clinicians' views of MI: Is it perceived as useful? Could it be integrated into practice? What training would be required to make it part of routine care? Nine respiratory clinicians attended a one-day MI workshop and a semi-structured face-to-face interview two weeks later. All interviews were audio-recorded, transcribed verbatim and analysed with thematic analysis. Four main themes are presented—1) MI’s suitability for use in respiratory contexts: participants saw potential in using MI to motivate their patients to engage with prescribed respiratory interventions, such as increased physical activity. Those who experimented with new skills post-workshop were encouraged by patient responsiveness and outcomes. 2) MI’s relationship with routine clinical practice: some believed they already used elements of MI, but most participants felt MI was fundamentally 'different' to their normal style of working. 3) Implementation issues: additional time would need to be made available to enable an appropriate depth of conversation. 4) Training issues: Participants sensed the complexity of MI could make it difficult to learn and that it would take them time to become competent. On-going supervision was perceived as necessary. One key challenge identified was how to suppress behaviours that are antithetical to MI. These findings lend support to the feasibility of using MI in respiratory contexts such as pulmonary rehabilitation programmes, but highlight implementation and training issues that would need to be overcome. The insights have informed the development of another study, testing the effect of a tailored training package on MI skill, specifically for respiratory clinicians delivering pulmonary rehabilitation programmes.

## Introduction

The success of therapy for many conditions is influenced by the extent to which patients engage with their treatment and adhere to the activities and lifestyle changes that are recommended to them. However, in the respiratory field, making such changes usually requires a high degree of effort and motivation, and poor adherence is common [1–3]. An important task for clinicians working in this area, therefore, is enhancing motivation for behaviour change. This paper provides insight into clinicians’ views of an approach to changing health behaviour (motivational interviewing) adapted specifically for respiratory patients.

Motivational interviewing (MI) has been defined as “a collaborative conversation style for strengthening a person’s own motivation and commitment to change” [4]. According to MI theory, unresolved ambivalence—the experience of being torn between conflicting, yet co-existing motives—is what is most likely to undermine sustained behaviour change [4]. Patients with respiratory conditions may be motivated to change their behaviour (mobilise, exercise more, stop smoking) to improve their health, but simultaneously lack confidence in their ability to succeed, or may perceive that the personal costs, such as the time and effort it takes, to be insurmountable. MI seeks to create a supportive, empathic atmosphere whilst gently encouraging patients towards thinking about and verbalising their own motivations for change, as well as considering how their current behaviour is influencing their ability to lead the life they ultimately want [5]. The aim is to help patients to explore and resolve their ambivalence.

Meta-analyses of MI across behaviours and contexts have shown that MI can be effective in encouraging behaviour change, and also efficient, often taking less clinician time to achieve change than comparison interventions [6–8]. The strongest evidence for MI comes from the addictions field, where it was originally developed; although there is emerging evidence for its utility in health contexts [6, 9, 10]. The motivation and adherence issues that present in respiratory contexts closely resemble those where MI has been shown to be useful, which is why there is currently some interest in using it to help patients with respiratory conditions [11–13]. Although there is some evidence for this application of MI, the work tends to be limited by small sample sizes and insufficient reporting of treatment fidelity [14–19]. To be considered a genuine test of MI, trials need to: 1) confirm that the intended intervention contains all key elements of MI (engaging, focusing and evoking) rather than simply stating it was informed by, or contained strategies derived from MI; 2) demonstrate that interventionists had met specific proficiency criteria prior to the trial; 3) report the integrity of MI delivery during the trial; and 4) report the reliability of integrity measures used [20].

One particularly useful application of MI is where it is used in combination with other established interventions. When MI has been used prior to, or alongside, other established interventions, it has been reported that attendance, engagement and long-term outcome all improve [8, 21]. This suggests that one potential respiratory application of MI is to use it to extend and prolong the known beneficial effects of pulmonary rehabilitation (a programme comprising exercise and educational elements) for people with COPD.

Before recommending the transfer of MI into other contexts such as pulmonary rehabilitation, it is important to test its effectiveness within these patient groups. A necessary pre-requisite for designing a definitive trial in this area is to undertake qualitative work to identify the perceptions of the professionals who would be responsible for delivering the intervention in everyday practice [22, 23].

The aim of this study was to establish:

- do respiratory clinicians feel that MI is a useful intervention?
- do respiratory clinicians feel that MI could be integrated into their routine practice?
- do respiratory clinicians believe that they can deliver MI as intended?
- are there any training issues that respiratory clinicians foresee?
- what might prevent MI being delivered within clinical practice?

## Method

### Design

A qualitative design involving semi-structured interviews and thematic analysis was used to answer the research questions. We were interested in gathering respiratory clinicians’ attitudes, beliefs and feelings regarding MI, and therefore a qualitative design was appropriate. A semi-structured interview approach was selected because this gave the adequate balance between providing the structure and focus of the pre-determined interview schedule, with the flexibility of open-ended questions and use of follow-up questions and prompts [24]. As the aim of the study was to understand the individual experience of the participants, this was considered more appropriate than a focus group approach, which could have led to issues with sharing views in front of other participants, plus the logistics of arranging a group meeting for busy clinicians. Thematic analysis was selected, because it is a flexible approach with theoretical freedom, that can provide “a rich and detailed, yet complex, account of data” [24].

### Recruitment and participants

Following attendance at a one day ‘Introduction to Motivational Interviewing workshop’ run by the first author (RS), all attendees (n = 18) were given a study recruitment pack inviting them to take part in a qualitative study to explore their views of the use of MI within the respiratory setting (see S1 Table for details of the MI workshop). Those interested in participating were asked to make contact with a member of the research team (MD-H) to arrange a suitable time to obtain consent and undertake the interview. This resulted in a convenience sample of nine female clinicians (three physiotherapists, four nurses, one occupational therapist and one rehabilitation assistant) who worked in a respiratory service (in-patient and out-patient) within a community hospital in the South of England.

### Researcher background

In the service of transparency, we provide contextual information about the authors in this section, to enable the reader to make judgments about the how our backgrounds, pre-existing beliefs and experiences might have influenced the research [25]. The first author (RS) delivered the 1-day introductory MI workshop and with support, analysed the interview data and wrote the manuscript. He is a Chartered Sport and Exercise Psychologist who teaches healthcare communication (with a focus on encouraging health behaviour change) to allied health professionals and nurses at both pre and post registration levels within the Faculty of Health Sciences, University of Southampton. He has been delivering training in MI for over 20 years and is a member of the Motivational Interviewing Network of Trainers, an international organisation that aims to promote high quality practice and training. He embarked on the present study, therefore with a sound knowledge and experience of MI, but with some potential for bias in favour of its use. This research is the first of three studies that form his PhD, focused on the feasibility of MI for promoting adherence to physical activity recommendations as part of pulmonary rehabilitation. He is being supervised by the co-authors of this paper (MD-H & AB). MD-H is a Health Psychologist, Senior Lecturer, and experienced qualitative researcher. She interviewed the participants following the workshop and supported RS in the analysis of the interview data. AB is a Professor of Respiratory Rehabilitation with a clinical background in respiratory physiotherapy who leads Respiratory Research within the Faculty of Health Sciences, in which one of the key themes is adherence to therapy. She had input into the initial research design, and both she and MD-H have contributed to all drafts of the written report.

### Procedure

Ethics approval for the study was obtained from the National Health Service Integrated Research Application System (REC Reference Number: 06/Q1704/33). The second author (MD-H) interviewed each participant individually face-to-face after having gained written informed consent. She observed the workshop but was otherwise independent. The interviews were carried out approximately two weeks after the MI workshop, so participants had already had an opportunity to reflect on what they had learnt in the context of their routine practice. During the semi-structured interviews MD-H explored topics relating to 1) what the participants remembered from the workshop and their views about its content, 2) whether they thought they could implement the skills in their current practice, 3) what they thought were the key challenges in implementing the approach, and 4) what training needs they anticipated having in order to deliver motivational interviewing skilfully (please see S1 Text for interview schedule). The interviews lasted between 20 and 50 minutes and were carried out within a hospital setting. All interviews were audio-recorded and transcribed verbatim, however the transcripts were modified where necessary to preserve confidentiality and anonymity.

### Data analysis

A systematic approach to thematic analysis described by Braun and Clarke [24] and recommended by others as best practice [26] was adopted. The phases summarised in Table 1 were completed. These phases are recursive, meaning that it was necessary to return to earlier stages frequently, to ensure consistency between the themes and data.

**Table 1:** Phases of thematic analysis adapted from Braun and Clarke (2006) [24].

Phase	Description of Process
1. Data familiarisation	Audio-recordings of interviews were transcribed verbatim (RS). Transcripts were read and re-read (RS). Initial ideas were noted (RS)
2. Generating initial codes	Systematic coding of entire data set on a line-by-line basis was performed (codes were assigned to chunks of data—words, sentences or paragraphs) (RS). Extracts of data relevant to each code were collated (RS). Written reflection on interesting features was produced (RS).
3. Searching for themes	Excerpts from each code were re-read (RS). Codes were collated into potential themes (RS).
4. Reviewing themes	Research team members checked whether the themes worked in relation to the coded extracts (RS, MD-H). All transcripts were re-read to establish whether themes worked in relation to the data set (RS).
5. Defining and describing themes	On-going analysis was performed to refine the specifics of each theme and the overall narrative of the analysis (RS, MD-H). Research team members checked that each theme captured something important in relation to the overall research question.
6. Producing the report	Appropriate examples of extracts that best represent each theme/subtheme were selected (RS, MD-H, AB). The analysis was linked to the research questions and literature (RS, MD-H, AB). A final written report was produced (RS, MD-H, AB).

Analysis was not theory driven so codes for the emergent themes were developed inductively from the interview transcripts. In this sense they are closely linked to the participant narratives and the authors made a conscious effort to prevent any preconceived ideas and knowledge of MI from constraining the analysis. On-going reflexive dialogue within the team throughout the analytic process served to maximise the correspondence between participants’ description and the authors’ representation of them. Themes were identified primarily from the explicit content of participant interviews, though it was necessary to examine latent themes regarding participants’ perception of motivational interviewing in relation to normal practice. The sub-theme ‘It is quite foreign at first’, therefore drew on ideas and conceptualisations that were thought to underpin their views about the potential of MI in their work.

Strategies were employed to maximise the trustworthiness of the analysis and reporting [27]. The on-going reflexive dialogue within the team, mentioned above, helped to ensure adherence to the analytical method and minimise bias in the interpretation of the interview data. Constant comparison was used to systematically develop and refine the content of categories during coding and generation of themes. Finally, an audit trail was developed (available from corresponding author on request) detailing the decisions that were made together with their rationale and acknowledgment of the impact of any potential bias.

## Results

The findings are described under the four main themes that emerged from the data.

### Theme 1: Motivational interviewing’s suitability for use in respiratory contexts

Most participants talked favourably about the use of MI within the respiratory setting. They believed that MI improved the relationship that they had with patients and had the potential to be an effective method to motivate patients to change behaviour. Participants described it as a pragmatic approach that might be used in several situations in their clinical practice: smoking cessation, mobilising, exercise, reducing alcohol consumption and modifying diet.

*“…I could see and every example they gave just made so much sense*. *It’s um*, *it is something that I would like to pursue*.*”* [Participant 3, Physiotherapist]

The potential of MI to promote engagement and commitment to pulmonary rehabilitation was also discussed. Sometimes patients agree to attend for the programme but are at a high risk of drop out because at the time of committing to it, they do not appreciate the intensity of exercise that is involved or the requirement for additional physical activity between group sessions.

*“… but it’s so hard …*. *but they present themselves for a pre assessment session and they all come in and their whole body language is of ahh*, *what am I signing up for here*?*”* [Participant 2, Physiotherapist]

Another participant described how some patients are reticent about making changes in their physical activity because of a fear of breathlessness. The style of MI was perceived to be helpful in these circumstances.

*“Umm the other thing is exercise for our pulmonary rehab…*. *trying to encourage people that are breathless to exercise is really difficult but they are so scared of their breathlessness anyway*.*”* [Participant 4, Nurse]

Following the workshop, without prompting, several the clinicians tried out some of the skills they learnt about in their practice. One of the physiotherapists noticed greater responsiveness from patients whilst using MI as compared with her normal way of working.

*“Umm*, *but I have had a little bit of go on certain patients on the ward*. *And I think what I have done*, *it does*, *you see quite a different reaction from the patient*, *they seem to have a lot more belief in what you are trying to encourage them to do*.*”* [Participant 8, Physiotherapist]

Another participant, an Occupational Therapist described beneficial effects of MI which reinforced her belief in its effectiveness.

*“… I worked with a particular patient in a different way and had a very good outcome*, *umm which I was really—it was really positive and also another person on the course*, *the other physio*, *we worked together with this patient in the same way*, *umm and you could see it did work*.*”* [Participant 5, Occupational Therapist]

Reflective listening seemed to be an important new skill to clarify their understanding of what the patient had said. This helped to improve the quality of the relationship that they felt they had with the patient.

*“Not that I didn’t listen before but I think actually taken on board maybe more what they are saying and showing that I understand whereas before you might listened to a patient and then you say right okay that’s good let’s*, *now because I’m paraphrasing what they’re saying to me it shows them that I have actually listened to what they’ve said and I think you get a much better rapport that way with patients and they’re more willing to do what you want them to do*.*”* [Participant 8, Physiotherapist]

Underpinning these views was recognition that a change in the clinician’s practice behaviour leads to changes in how patients respond. For example, participants observed less passivity, reduced resistance and greater levels of motivation for change from patients.

*“Yeah we had a patient in who’d been in quite a while she was very good she could move quite well and she just would refuse a lot of the time*, *and I’d get frustrated*, *she’d get frustrated and it wasn’t*, *you know*, *it wasn’t bad but it was just both were getting frustrated and were going anywhere so I tried*, *myself and another colleague who did it and we tried the motivational interviewing …*. *we did get her to transfer really well and not and she was quite shaky before but this time she was a lot better*.*”* [Participant 9, Physiotherapist]

Arguably the strongest support for its use came from a nurse participant who valued it so much that she was concerned that she might relapse back to old ways of working. Here she refers to her righting reflex—the natural helping inclination to provide advice at the expense of evoking the patient’s ideas, which has the potential to trigger resistance and undermine engagement.

*“I just hope I continue to use and that my own reflex*, *righting reflex*, *and my own previous assessment techniques don’t just slip back in as a bad habit*, *you know*, *I am worried that I will get one tired week or one bad week and start going back to how I was before*.*”* [Participant 4, Nurse]

### Theme 2: Motivational interviewing’s relationship with routine clinical practice

#### “I do this already”

Whilst MI represented a novel way of working for most participants within this sample, some believed that they were already using elements of the approach, prior to the workshop.

*“But I think I do I do have aspects of motivational interviewing in me anyway when I talk*, *when I listen…”* [Participant 9, Physiotherapist]

One participant felt that her previous training in counselling, combined with her clinical experience meant that she had developed her own way of consulting with patients about behaviour change. When questioned on whether the workshop had prompted any changes in her practice she said:

*“I’m going to have to be very frank here and say not an exceptional amount because I’ve been dealing with them for a long time*, *I did a counselling course some years ago and that taught me quite a lot*.*”* [Participant 2, Physiotherapist]

Another participant described the workshop as having consolidated many of the skills that she had developed instinctively.

*“Yeah I did feel that I was doing most of it anyway*. *But for somebody to tell me that this is what we do and this is why we are doing it*. *And I thought oh*, *actually that makes perfect sense yeah*.*”* [Participant 1, Nurse]

We had anticipated that some participants might perceive similarities between MI and their routine clinical practice. MI has been described as a refined form of guiding that is patient-centred and draws on skills such as asking, informing and listening that are often used in everyday practice [28]. On first exposure, MI can appear deceptively simple; a fluid conversation in which the patient is engaged and becoming increasingly committed to the change side of their ambivalence. Some of the skills may be familiar to an observer, though the strategy underpinning the specific way they are used may not be so obvious [29]. Monitoring and responding to patient reaction on a moment-by-moment basis, attending to the unspoken meaning of what the patient has expressed, as well as arranging the conversation in a way that encourages the patient to explore and resolve their ambivalence, are examples of MI components that are not immediately apparent and are difficult to discern following a brief workshop.

When explaining which elements of MI they felt they were already using in their practice, most described a general patient-centred orientation, rather than anything that is unique to MI. This suggests that the workshop had not been successful in helping these participants make sense of all the elements that make MI different:

*“Well I always go on the patient’s past history and what they prefer and how they would like to be cared prior to coming into hospital*. *I always try and research that from the carer and the patient as to what their habits were*, *when did they try different things before*, *umm to try and change their behaviour*.*”* [Participant 7, Nurse]

One participant expressed uncertainty as to whether she was using MI or not, and described the open nature of her consulting style:

*“Yeah*, *probably not in the way it was completely phrased or* [workshop facilitator] *put it across but yes*, *I think*, *and it’s personal that I ask too many closed questions and not open questions but I do allow the patient to talk quite openly and freely*.*”* [Participant 2, Physiotherapist]

Whilst the above descriptions are compatible with MI, they do not reflect the refined use of the skills, or the underpinning strategy that distinguishes MI from everyday patient-centred conversations. The comments suggest an oversimplified picture was presented, that resulted in some participants believing they were already using MI.

#### “It is quite foreign at first”

Many participants did recognise differences between what was taught in the workshop and what they normally deliver in practice. One participant lamented her reliance on advice giving:

*“Um*, *I’d still listen*, *but I don’t think I’d probably*, *I’d probably more bombard them with advice and say look it’s important you do this because of this…”* [Participant 9, Physiotherapist]

Others spoke about the need to think about the specific language that they use and the benefit of evoking the patient’s perception of their problem.

*“I remember thinking at the time I am definitely going to do that*. *I can see there’s a genuine benefit of just*, *you know letting the patient tell you what the problem is*.*”* [Participant 1, Nurse]

Although MI draws on some familiar skills, most of the participants believed MI to be fundamentally 'different' to their current style of working.

“*But I found just the whole way of posing the questions and being open and maybe a little bit more um I don’t know*, *non-judgemental about your approach to interviewing with a patient*.*”* [Participant 3, Physiotherapist]

For the majority, the philosophy and practice of MI was perceived as antithetical to their normal way of encouraging behaviour change. It calls for a re-direction of their ‘expert role’ from one in which the aim is to install motivation through the provision of expert advice, to one which relies on empathic counselling skills to carefully draw out and reinforce the patient’s own reasons for making changes.

*“… I thought before that when they had been to their GP*, *the GPs probably waved a finger and said you most give up smoking it is bad and so when they come into me because I try and be friendly and nice about I feel that I was doing a better*, *but I was actually doing the same thing just in a different tone of voice*.*”* [Participant 4, Nurse]

Practicing MI, therefore, often necessitates the suppression of tacit, over-learned counselling habits that run counter to its collaborative, evocative and autonomy respecting style. This need is reflected in the following quote from participant 4:

*“I think the main thing was we kind of all discovered as healthcare professionals that our righting reflex was really strong and when we were trying to do the role plays we were automatically trying to correct what people were saying*, *they were saying ‘oh I can’t do this’ and we were saying ‘yes you can you can this because this that and the other’*.*”* [Participant 4, Nurse]

Although participants had previously experienced some success with advice giving to encourage behaviour change, with sufficient success to reinforce this style of working, they also thought its effectiveness was limited. Furthermore, they voiced concerns regarding its potential for undermining rapport and increasing patient defensiveness.

*“.. don’t just force your*, *not your opinions*, *but the health information on someone because sometimes they’ve heard it so many times before like the smokers and things and it’s trying to work out why it hasn’t worked rather than just bombarding them with it because it’s not going to have any effect they’ll just deflect it like they have with any other professional*.*”* [Participant 9, Physiotherapist]

Another difference between MI and routine practice that participants discussed was the proportion of talk time occupied by the clinician and patient. Participant 4 discussed how she had previously viewed silence as a cue to say more, and thus would dominate the consultation. Using skills such as silence to encourage involvement was novel and felt awkward, but was viewed positively.

*“It is getting more comfortable*, *it is quite foreign at first*, *particularly just not talking…*. *because I think particularly my own personality problem is the fact that there is a silence I will try and fill it in and if there is a patient in front of me I think that if there is silence they’re asking me to tell them something so I’ll then continue to give them more and more advice*, *so just sitting back and letting there be a silence*, *does mean that actually they will generally start talking themselves and continue to talk about their areas*.*”* [Participant 4, Nurse].

Most participants indicated that they reflected on their normal consulting style as a result of the introductory workshop. One in particular evaluated it quite negatively:

*“I am just information giving yeah continuously*, *it makes me sound like a bully*, *I don’t think it is bullying at the time*.*”* [Participant 4, Nurse]

Although an awareness of the difference between the style and focus of MI and routine practice maybe a precondition for learning the method, this quote suggests that being introduced to MI and trying out the skills with patients can be somewhat destabilising. Another physiotherapist indicated that she uses closed questions almost exclusively in consultations.

*“As physios we maybe used to expecting an answer and almost give a prompt to the patient in your questioning we give a prompt rather than allow them freely to give the information over to you*. *Pretty well the clearest things* [from the workshop training] *were how you question patients and how you phrase rather than standard closed questioning that physios I think preferentially tend to use*. *You know we’ve got black and white answers to the questions we’re asking”* [Participant 2, Physiotherapist]

For this participant, closed questions help her to maintain control of the consultation. This prevents the discussion from straying into psychosocial matters, which were deemed to be ambiguous and more difficult to address. Doing so serves a practical function, in ensuring that the consultation keeps to time, but also a protective one for the therapist because it pre-empts the need to address what might be emotive issues that they feel less equipped to address.

*“So it may be just phrased in a different way rather than having the confidence to let it be a more open discussion… They should be equal*, *[physical and psychosocial issues] that’s the problem that we*, *you know*, *maybe being physiotherapists you always see it as the physical because that’s the way that you are naturally trained”* [Participant 2, Physiotherapist]

### Theme 3: Implementation issues

In general, these participants viewed MI positively. However, they also expressed some issues regarding its integration within routine clinical practice. The pace of modern healthcare is fast, with many pressures that compete for clinicians’ time; a lack of time was felt to be a significant factor that might present a barrier to its use.

*“It’s quite hard when the focus for us on the medical wards is to get people fit enough to leave the hospital as quick as possible*. *So it’s you know I’m doing this if you like on the back of a lot of what we’re you know we’re very restricted really on the time that we have for patients*.*”* [Participant 3, Physiotherapist]

On the other hand, there was a sense that some additional investment in time (particularly listening) might actually lead to greater levels of overall efficiency in the care that is provided.

*“… you don’t have loads of time with patients um and I find that you just tend to go in*, *give them all the information or whatever and then get out*, *rather than spending a more time sometimes spending more time with them initially–just an extra 10 minutes means you don’t need to spend so much time with them later on*.*”* [Participant 9, Physiotherapist].

When participants reflected on what is important from the patients’ perspective, taking time to listen and to respond to the patient’s needs and priorities was seen to be a worthwhile investment.

*“… the nurses are rushing them to do this*, *the physios are rushing to do something and everybody is at them and then suddenly there is somebody who has actually got a little bit of time for them and it seems to make a huge difference ‘oh you give me so much confidence’ and that’s really nice for me to hear*.*”* [Participant 6, Rehabilitation Assistant]

Clinician participants were keen to impress that they have several different tasks to undertake as part of their role, which may demand different styles of communication. Whilst MI was perceived useful for addressing behaviour change issues, a key to its integration would be the need to switch flexibly between different communication styles, based on the situation. One participant discussed this in the context of an initial consultation prior to pulmonary rehabilitation, involving a combination of instruction, assessment and discussion about helpful behaviour change.

*“…*. *there is definitely a place for motivation interviewing to discuss certain areas*, *say about their smoking or about their exercise*, *but then the other half of the interview we do need to give advice about their medication and when is best to take it and umm what other factors to avoid to make their chest worse*, *so we have to be kind of clever in the way*, *I think that we need to incorporate both of those techniques*…*”* [Participant 4, Nurse)

Two important considerations expressed were the timing of MI interaction, and the need for a quiet space. This was relevant, particularly on busy wards where the clinicians felt conscious that the content of what might be discussed would necessitate additional privacy not afforded by the setting.

*“The pressures of the staffing levels and um the general sort of ambiance on the ward you can often perhaps want to spend some time in quiet with a patient and you have to make a real effort to find both of those—the time and the quiet to do it”* [Participant 3, Physiotherapist]

### Theme 4: Training issues

Whilst these participants had experienced some success with their efforts to implement MI, they also acknowledged that it would take time and practice to develop proficiency. One particular challenge was felt to be the use of empathic listening. This is a fundamental skill that involves listening carefully to what the patient is saying, and using reflective listening statements to clarify understanding. Reflections vary in depth with the content of simple reflections staying close to what the patient has stated, whilst more complex reflections capture the underlying meaning (what has been implied but not stated explicitly) [4]. As one might imagine, being able to use complex reflections to differentially evoke and reinforce patient’s own motivations for change (a characteristic of more advanced MI practice) is a novel skill which was demanding for these participants.

*“… it was just that actually trying*, *rather than just repeat what the other person is saying*, *actually trying either paraphrase or emotionally reflect what they are saying*, *I found really difficult and I think is one of those skills that you really need to practice to improve on really*.*”* [Participant 8, Physiotherapist]

The participants had attended a one-day training in MI, and the consensus was that whilst this was sufficient to create interest and promote enough insight and confidence to try it in practice, additional tailored training with coaching and feedback would be required to promote proficiency.

*“… I think the day is enough for you to pick it up and understand what it’s about but it’s the practice that*, *improves …so having the feedback when you have seen a real patient and applied it would be useful*.*”* [Participant 9, Physiotherapist]

## Discussion

Motivational interviewing is a method for resolving the common problem of ambivalence about behaviour change [4]. Although the strongest evidence of effectiveness is in the addictions field, there is considerable interest in applying the same principles to help patients with respiratory problems, for whom effective self-management is known to be of benefit [30]. The results of this study lend support to the feasibility for the use of MI in respiratory care, but raises issues surrounding the desirability for further training, beyond a one-day workshop.

This work is novel in its focus on the feasibility of pure MI rather than a derivative of the pristine method, which is a common feature when attempts are made to apply MI to health contexts. Using adapted forms of MI may be time-efficient in terms of intervention delivery and may reduce training requirements, but runs the risk that intervention ‘failure’ may be due to a lack of fidelity to the original format, rather than a failure of MI [20]. This study has also provided some preliminary insights into clinicians’ views of MI used as an adjunct to pulmonary rehabilitation to enhance engagement and adherence. Identifying ways to extend and prolong the beneficial effects of pulmonary rehabilitation has been identified as a key research question [31] and studies of interventions to encourage uptake and adherence to PR are now seen as a research priority [32]. One of the strengths of this research is that it involved clinicians from a range of different professional groups, and with different levels of experience of respiratory therapy. To date, most published work applying MI to respiratory contexts has only involved nurses. In the UK, physiotherapists are often the primary point of contact for respiratory patients referred for pulmonary rehabilitation, and are therefore frequently key to encouraging any behaviour change.

The thematic analysis used in this research was primarily inductive, in that the themes were derived mainly from the participant narratives, rather than being aligned to a particular theoretical position. We are aware that prior exposure to MI training and implementation literature will have inevitably influenced our interpretations. As Braun and Clarke note, however, it is not possible for researchers to divorce themselves from pre-existing knowledge [24]. Workshop participants were informed prior to consenting to join the study that the first author (RS) who delivered the MI workshop would also be involved in the analysis of interview data. Those with more negative perceptions of the workshop and of the utility of MI may have been dissuaded from participating to avoid offence or personal embarrassment. Equally, those who elected to participate may have felt inclined to share perceptions that were more complimentary and endorsed the approach. However, neither MD-H nor AB had prior experience of MI, or held any particular views about it

The one-day workshop delivered in this study was designed to introduce the critical elements and theoretical underpinnings of MI, and help participants appreciate how it differs from traditional practice. Another goal was to avoid alienating participants by providing so much detail that they left the workshop confused and/or believing they would never be able to master the skills involved. Whilst it was not the original intention, some attendees were sufficiently motivated and had enough confidence to try out what they had learnt in practice. These participants were encouraged by the responsiveness of their patients and the outcomes they achieved with the approach. However, as participants themselves admitted, a one-day workshop was not sufficient to promote proficiency in the method. This experience is consistent with conclusions from meta-analyses of MI training across other professional groups, that emphasise the importance of additional training in the form of coaching and feedback on practice [33, 34].

It is not yet known how much MI training respiratory clinicians would need in order to develop a level of skill that is capable of making a difference to long-term patient outcomes. The insights gained here have informed the development of a study testing the effect of a tailored training package on MI skill, specifically designed for clinicians delivering pulmonary rehabilitation. There is some evidence from the studies in the addictions field that prior counselling skills (such as accurate empathy, which is foundational to MI) may influence overall MI skill acquisition and retention [35, 36] and a plausible model to explain this observation has been proposed [37]. This means that respiratory clinicians who typically do not have prior training in counselling may find it harder to learn MI than psychologists and specialist counsellors, for whom MI training represents a refocusing of previously attained skills, a point that has been raised by others [5]. For the participants in this study, learning to do MI would involve not only developing a new set of skills, but also de-automating and learning to supress ways of talking with patients that are incompatible with MI. Training would therefore need to focus on both of these issues. Insights gained from this study indicate the importance of teaching respiratory clinicians how to address emotive issues that may emerge during MI, and the need for some flexibility in consultation duration, at least at the outset, while skills are new and relatively inefficient. For these reasons, any future trials of MI should incorporate a cost-benefit analysis that includes the training component for those providing the intervention.

The themes presented here reflect the perceptions of clinicians who were sufficiently interested in MI to attend a workshop and interview, so other clinicians may hold less positive views of the utility of MI. Despite this caveat, we believe our findings should be transferable to many clinicians working in this type of setting.

## Conclusion

These findings lend support to the feasibility of using motivational interviewing (MI) in respiratory contexts, such as pulmonary rehabilitation programmes, but highlight potential implementation and training issues for respiratory clinicians who wish to deliver MI effectively. The style of a MI encounter is fundamentally different from traditional approaches to working with patients, but this is not always immediately grasped by those learning it during short training courses. On-going training and additional contact time are necessary to become proficient at the use of MI. These respiratory clinicians viewed MI positively and noted increased patient responsiveness when trying out skills in practice, but also perceived that more training time would be needed to enable them to embed MI within their practice.

## Supporting information

S1 Table (Workshop: Introduction to motivational interviewing in respiratory contexts). (DOCX)

S1 Text (Semi-structured interview guide). (DOCX)
