# Supplementary material for: Motivational interviewing in respiratory therapy: What do clinicians need to make it part of routine care? A qualitative study
Source: PLoS One. 2017 Oct 31;12(10):e0187335. doi: 10.1371/journal.pone.0187335 (PMC5663487; doi:10.1371/journal.pone.0187335)
Supplement: S1 Table — (DOCX) [file pone.0187335.s001.docx]

**Workshop: Introduction to motivational interviewing in respiratory contexts**

**Workshop aim:** to equip participants with a basic knowledge of the spirit and processes of motivational interviewing (MI) and provide opportunities to practise some of the key skills.

**Workshop learning outcomes**:

1. Describe an MI conceptualisation of engagement and adherence issues in respiratory contexts
2. Describe the fundamental spirit and processes of MI
3. Provide opportunities to practise empathic counselling skills
4. To understand and practise some of the directive aspects of MI that are relevant to behaviour change discussions in respiratory contexts
5. Describe the patient language cues (“change talk” and “resistance”) that provide continued feedback and enable continuous learning in practice.

**Workshop schedule**

| **Type** | **Subject** | **Focus** | **Timing** |
| --- | --- | --- | --- |
| *Discussion* | Workshop introduction | Introductions, aims and learning outcomes, participant expectations and hopes | 20 mins |
| *Mini-lecture* | Background  Philosophical, conceptual issues | Common assumptions about why people don’t adopt healthy lifestyles. MI conceptualisation of engagement and adherence issues in respiratory contexts (ambivalence: *’I know I should, but I don’t want to’*) | 30 mins |
| *Experiential Exercises* | Contrasting deficit versus competence perspectives on motivation | Contrasting ways to handle patients’ ambivalence – advice giving/persuasion versus listening and evoking. Exercise #1 Persuasion role play. Feedback of patient and clinician’s experiences in plenary  Discussion: why do people resist our good advice? Righting reflex, resistance, the need to be understood and accepted, the significance of patient language. Preparation for exercise #2. Recognising change talk: brief introduction followed by ‘drumming for change exercise’. Empathic listening: Using reflective listening to convey empathy and to develop the patient’s own arguments for change. Brief introduction. Exercise #2 Listening and evoking real play. Feedback of speakers’ and listeners’ experiences in plenary. Discussion: contrasting approaches. | 60 mins |
| **Break** |  |  | 20 mins |
| *Mini-lecture* | MI framework: definition, spirit & processes | Overview of the aims, overall style of working, principles of practice and processes involved. | 20 mins |
| *Video analysis* | MI-consistent practice | Participants watch video focusing on overall style of working, skills involved and change talk that emerged. Discussion: Feedback on observations. | 30 mins |
| **Lunch** |  |  | 45mins |
| *Mini-lecture* | Core skills | Core skills that underpin conversations about behaviour change: Asking good open-ended questions; Affirming – noticing and commenting on effort, strengths, determination; reflective listening to convey empathy; summarising the discussion. | 15mins |
| *Skill building exercises* | Core skills | Question quiz. Reflective listening exercise i) thinking reflectively -hypothesizing meaning ii) forming reflections iii) levels of reflection. | 45 mins |
| **Break** |  |  | 20 mins |
| *Mini-lecture, demonstration, followed by skill building role-play/real-play* | Example strategies | i) Raising the subject of behaviour change/setting the scene and engaging using the typical day strategy. ii) Using evoking skills to build importance and confidence for change. Identifying, elaborating and reinforcing talk about changing behaviour. iii) Sharing information in an MI style | 60 mins |
| *Mini-lecture followed by skill building exercise* | Responding to resistance | Minimising defensiveness and responding to it when it does occur. | 30 mins |
| *Discussion* | Reflection | Participants feedback on their experience of MI  Discussion regarding potential implications for practice. | 15 mins |
| **Finish** |  |  |  |
