# Supplementary material for: Motivational interviewing in respiratory therapy: What do clinicians need to make it part of routine care? A qualitative study
Source: PLoS One. 2017 Oct 31;12(10):e0187335. doi: 10.1371/journal.pone.0187335 (PMC5663487; doi:10.1371/journal.pone.0187335)
Supplement: S1 Text — (DOCX) [file pone.0187335.s002.docx]

**Semi-Structured Interview Guide**

1. What are the key things that you remember from the workshop?
2. What do you consider to be the strengths of the approach in relation to the work that you do?
3. What do you see as its limitations?
4. In what way (if at all) has it influenced the way you think about or approach behaviour change conversations with your patients?
5. To what extent do you feel that motivational interviewing should be integrated into routine practice within respiratory care?
6. What do you think would be the challenges to your adopting this approach and delivering it within your practice?
7. What training requirements do you foresee as necessary to enable you to deliver motivational interviewing effectively?
